# Pet husbandry and infection control practices related to zoonotic disease risks in Ontario, Canada

**DOI:** 10.1186/1471-2458-13-520

**Published:** 2013-05-29

**Authors:** Jason W Stull, Andrew S Peregrine, Jan M Sargeant, J Scott Weese

**Affiliations:** 1 Department of Pathobiology, Ontario Veterinary College, University of Guelph, Guelph, Ontario, N1G2W1 Canada; 2 Centre for Public Health and Zoonoses, University of Guelph, Guelph, Ontario N1G2W1, Canada; 3 Department of Population Medicine, Ontario Veterinary College, University of Guelph, Guelph, Ontario N1G2W1 Canada; 4 Current address: Department of Veterinary Preventive Medicine, The Ohio State University, College of Veterinary Medicine, 1920 Coffey Rd., Columbus, OH 43210, USA

**Keywords:** Zoonoses, Pet, Education, Infection, Husbandry, Canada

## Abstract

**Background:**

Many human infections are transmitted through contact with animals (zoonoses), including household pets. Despite this concern, there is limited knowledge of the public’s pet husbandry and infection control practices. The objective of this study was to characterize zoonotic disease related-husbandry and infection preventive practices in pet-owning households in Ontario, Canada.

**Methods:**

A self-administered questionnaire was distributed to individuals at two multi-physician clinics in Waterloo, Ontario, Canada during 2010. One adult from each household was invited to participate in the study.

**Results:**

Four hundred one pet-owners completed the questionnaire. Households reported ownership of dogs (68%), cats (48%), fish (13%), exotic mammals (7%), such as hamsters, and reptiles and birds (each 6%). Across all species, individuals at higher risk of infections (i.e. < 5yrs, ≥ 65yrs, immunocompromised) were often (46-57%) present in households. Children < 16 yrs of age had close pet contact, as households reported dogs (13%) and cats (30%) usually slept in a child’s bed and dogs often licked a child’s face (24%). Household husbandry practices that increase zoonotic disease risk were frequently identified; some fed high-risk foods (i.e. raw eggs, raw meat, or raw animal product treats) to their dogs (28%) or cats (3%); 14% of reptile-owning households allowed the pet to roam through the kitchen or washed it in the kitchen sink. Reported hand washing by children was high for all species (> 76% washed hands sometimes or greater after touching the pet, its feces, or housing), although fewer reported children always washed their hands (3-57%; by species). With a few exceptions, practices were not associated with the presence of higher risk members in the household or recall of having previously received zoonotic disease education.

**Conclusions:**

The results suggest there is a need for education on zoonotic disease prevention practices for pet-owning households with individuals at higher risk of infection and those with high-risk species (e.g., reptiles). Further research is needed to determine the role of education in altering higher risk pet practices.

## Background

Pet ownership is common, with the majority of households in many developed countries owning pets [1-8]. A recent study estimated that 56% of Canadian homes have at least one dog or cat, with a minority owning fish (12%), birds (5%), rabbits or hamsters (each 2%), lizards, guinea pigs, snakes, frogs, turtles, ferrets, or gerbils (each 1%) [6]. Pet ownership has been shown to have mental and physical benefits [9], particularly among children, the elderly and immunocompromised individuals [10-16]. However, despite these benefits, there are also potential health hazards associated with pet ownership and contact.

A recent estimate suggests that 14% of all human illness caused by seven common enteric pathogen groups (i.e., *Campylobacter* spp, *Cryptosporidium* spp, Shiga toxin–producing *Escherichia coli* (STEC) O157, STEC non-O157, *Listeria monocytogenes*, nontyphoidal *Salmonella* spp., and *Yersinia enterocolitica*) are attributable to animal contact [17]. Although the proportion of human disease for which pets are specifically responsible is largely unknown, greater than 70 pathogens of companion animals are known to be transmissible to people [18] and pet contact has been clearly established as a risk factor for illness caused by various pathogens, including *Campylobacter*[19,20], *Salmonella*[21], *Pasteurella multocida*[22] and *Capnocytophaga canimorsus*[23]. For some pet species, their role in human disease has been more clearly identified; reptiles and amphibians are estimated to be responsible for 6% of all sporadic *Salmonella* infections (11% among persons less than 21 yrs old) [24]. The emergence and dissemination of multidrug-resistant bacteria in both humans and companion animals has created additional concerns [25,26].

People can acquire pet-associated zoonotic organisms through the skin and mucous membranes (via animal bites, scratches, or direct or indirect contact with animal saliva, urine and other body fluids or secretions), ingestion of animal fecal material, inhalation of infectious aerosols or droplets, and through arthropods or other invertebrate vectors [27]. Although any exposed person can become infected with a zoonotic pathogen, risks are particularly high for those with a compromised or incompletely developed immune system, such as the young (< 5 yrs), elderly (≥ 65 yrs), pregnant and those with immune function-reducing conditions or treatments (e.g., diabetes, cancer, infection with human immunodeficiency virus (HIV), chemotherapy) [27,28].

Although incompletely understood, there are many factors that are involved in the occurrence of a zoonotic infection from a pet. Several of these factors likely have important roles in determining if an individual becomes infected, becomes symptomatic once infected, and the degree of illness that occurs. These include: 1) level and dose of exposure to the pathogen, 2) ability of the individual to suppress infection, with immunocompromised and extremes of age less able to successfully respond immunologically to pathogens, and 3) preventative veterinary care, husbandry and infection control practices that remove or reduce the level of the pathogen present and risk of transmission [27]. Existing recommendations stress that those at greatest risk for infectious disease should take the greatest precautions when interacting with pets, and be the most diligent in following infection prevention practices [29-35].

Despite the risk for pet-associated disease in people and role that appropriate infection control and husbandry practices may play in reducing disease risk, the area of household pet husbandry and infection practices has not been thoroughly investigated. The studies that have examined these topics, have noted the frequency of close contact between pets and people (e.g., licking of hands and sleeping in household member beds [36,37]), general pet ownership patterns by individuals at higher risk of disease [15,38-43], and general husbandry and infection control practices within pet-owning households (e.g., frequency of preventive veterinary care [40,44], and occurrence of hand hygiene [36,37] and husbandry practices [36,45]). However, previous studies have not analyzed the associations between these factors, which would allow for a better understanding of how the factors may change with differing household disease risks or with prior receipt of zoonotic disease education. The objective of this study was therefore to characterize pet husbandry and infection control practices that relate to zoonotic disease risks in pet-owning households in Ontario, Canada. Additionally, the study aimed to integrate household demographics, including human disease risk status (i.e., extremes of age, immunocompromised) and prior receipt of zoonotic disease education to determine if these factors influenced household practices.

## Methods

### Study location and selection of study sites

The site chosen for this study was the region of Waterloo, located in southern Ontario, Canada. The Waterloo Region is composed of three urban and four rural municipalities. This region was selected as it includes both rural and urban settings and its population has similar demographics to the Province of Ontario and Canada as a whole [46]. As of 2006, this region had a population of approximately 478,000 people in 178,000 private households. The median age was 36.4 yrs, with 81% of the population greater than 14 yrs. Median household annual income was $65,000 CAD [46]. These statistics are similar to those for the Province of Ontario and all of Canada, with the exception of a higher household median annual income ($60,000 for Ontario, $54,000 for Canada) [46].

Medical clinic and participant enrolment was part of a previously reported study that addressed knowledge and attitudes related to pet contact and associated zoonoses. Detailed enrolment methods and inclusion criteria are available elsewhere [47]. In brief, a convenience sample of multi-doctor general practice physician offices located within the Waterloo region was approached to participate in the study. Two practices agreed to participate (clinic A in Kitchener, Ontario and clinic B in Cambridge, Ontario).

### Data collection

During October and November, 2010, adults present in the waiting areas of the participating clinics were individually approached and invited to take part in the study. Individuals were eligible to participate if they were at least 18 yrs of age, able to read and speak English, and no one from their household had previously taken part in the study. When more than one eligible adult for a given household was present in the waiting area, a single household volunteer was requested. Participants were asked to complete an anonymous, confidential, self-administered written general questionnaire. Individuals who currently had pets in their household were also given a self-administered written 5-minute supplemental pet-specific questionnaire (Additional file 1). Household pets were defined as those that were indoor only, outdoor only, and those that spent time both indoor and outdoor. No incentives were offered for participating in the study.

The 7-page supplemental pet-specific questionnaire for this study was developed with guidance from epidemiologists, veterinarians, physicians and zoonotic disease experts. The questionnaire was pre-tested on six members of the public with varying zoonotic disease backgrounds, and revised accordingly. The final questionnaire utilized closed-ended questions, primarily Yes/No or multiple choice (using an ordinal scale). It gathered household-level data on the number and species of pets, pet husbandry and preventive medicine practices, involvement of children less than 16 yrs of age in select husbandry and pet-contact practices, childrens’ utilization of infection control practices, and the household’s and childrens’ emotional attachment to pets. Given the complexity of capturing and assessing flea and endoparasite prevention programs for each household pet, respondents were asked if all household dogs/cats were on intestinal parasite and flea prevention programs, without further defining, verifying or assessing specific components of the programs. Individual supplemental questionnaires were linked by respondent to the general questionnaire [47], allowing select variables collected through the general questionnaire to be utilized for additional analysis in this study. The study was approved by the University of Guelph’s Research Ethics Board.

### Data analysis

Data were entered into an Access database (Microsoft Corp., Redmond, WA, USA) and analyzed using Intercooled Stata version 10.1 (Stata Corp., College Station, TX, USA). Descriptive statistics were computed for all variables. Blank and “don’t know” responses were excluded from analyses. Household demographic and health data were extracted from the general questionnaire [47] to characterize households as higher risk [one or more individuals < 5 yrs, ≥ 65 yrs, or ever diagnosed with an immunocompromising condition (such as HIV/acquired immunodeficiency syndrome (AIDS), diabetes, cancer)] or not higher risk. Data regarding the respondents’ recall of having previously received pet-associated zoonotic disease education (i.e. having ever received information from any source about diseases that can be transmitted from pets or precautions to take with pets) were also extracted from the general questionnaire [47]. For statistical analysis, ordinal levels of each pet husbandry and infection control practice were collapsed to produce dichotomous outcome variables; decisions on how categories were collapsed were made *a priori* and based on existing recommendations for higher risk households and relevance of comparisons (Table 1) [29-35]. Statistical associations were individually assessed between pet husbandry and infection control practices (outcomes) and 1) households with individuals at higher risk of infections [at least one individual < 5 yrs, ≥ 65 yrs, or immunocompromised (exposed) vs. no one fulfilling these criteria (unexposed)] and 2) receipt of zoonotic disease education [previous receipt of pet-associated zoonotic disease education (exposed) vs. no recollection of having received this information (unexposed)]; Table 1. For households with children under 16 yrs of age, statistical associations were individually assessed between child-specific husbandry and infection control practices (e.g., child’s involvement and precautions taken in cleaning pet’s cages or feces, level of contact with pets) and households with all children at higher risk of infectious disease [presence of all household children < 5 yrs (exposed) vs. presence of all household children ≥ 5 yrs (unexposed)]. Due to small sample sizes for many of the variables, associations were measured using univariate exact logistic regression with conditional maximum likelihood estimates (CMLEs). When CMLEs were computed to be infinite, median unbiased estimates were calculated [48]. Odds ratios (ORs) and 95% confidence intervals (CI) for ORs were used to estimate the strength of associations. Statistical significance was based on a *P*-value < 0.05.

**Table 1:** **Outcomes related to animal husbandry and infection control practices tested for association with presence of higher risk individuals in the household and receipt of zoonotic disease education** (**Ontario**, **Canada**)


***I. Outcome variables (yes/no) tested for association with presence of any higher risk individuals in the household and prior receipt of zoonotic disease education***^***1***^	
Any uncontrolled outdoor access (cat)^2^	Dump aquarium water in bathtub, shower, kitchen sink, or bathroom sink (fish)
When outdoors, all always in fenced yard or on leash (dog)	Household very attached (cat, dog, reptile, fish, bird, exotic mammal)
Within past 3 months, any dogs on a leash at a dog park	Any fed raw animal product (cat, dog)^4^
Within past 3 months, any dogs off leash at a dog park (dog)	Any fed raw animal product not including raw animal treats (dog)
All on a flea prevention program (cat, dog)^3^	Any drink from the toilet (cat, dog)
All with outside access on flea prevention program (cat)	Any fed in the kitchen (cat, dog)
All on an intestinal parasite prevention program (cat, dog)^3^	Any used for breeding (dog)
All with outside access on an intestinal parasite prevention program (cat)	Any used for hunting (dog)
All have at least annual veterinary examinations (cat, dog)	Any declawed (cat)
Any allowed to roam freely through house (reptile)	Any ever bitten anyone (cat, dog)
Any allowed to roam in kitchen (reptile)	Any ever scratched anyone (cat)
Any cages or animals washed in bathtub or bathroom sink (reptile)	Clean litter boxes daily (cat)
Cage or litter boxes cleaned every several days, or more frequently (cat, reptile, bird, exotic mammal)	
***II. Outcome variables (yes/no) tested for association with all household children being higher risk (less than 5 yrs) and prior receipt of zoonotic disease education***^***1,5***^	
Any children have access to the litter box (cat)	Clean up feces immediately (dog)
Any children clean-up feces (cat, dog)	Never sleep in children’s bed (cat, dog)
Any children wear gloves when cleaning feces (dog)^6^	Any children play in same area pet goes to the bathroom (dog)
Children wash hands after cleaning feces (dog)^6^	Any children touch pet (cat, dog)
Ever lick children’s faces (dog)	Children never wash hands after touching pet (cat, dog)

^1^Presence of higher risk individuals in the household defined as one or more individuals < 5 yrs, ≥ 65 yrs, or ever diagnosed with an immunocompromising condition (e.g., HIV/AIDS, diabetes, cancer). Receipt of zoonotic disease information defined as recollection of having ever received information from any source about diseases that can be transmitted from pets or precautions to take with pets.

^2^Uncontrolled outdoor access defined as households with one or more outdoor only cats or indoor/outdoor cats not placed on a leash when outdoors.

^3^Respondant asked if all dogs/cats on flea or endoparasite prevention programs; the specific components of the programs were not assessed.

^4^Raw animal products defined as raw meat, raw eggs, or raw animal product treats.

^5^Analysis investigating the presence of higher risk children in the household was only performed for children-specific outcomes and only concerned comparison between children-containing households in which all children were < 5 yrs and those in which all children were ≥ 5 yrs.

^6^Association only assessed with prior receipt of zoonotic disease education.

## Results

Six hundred forty one of the 853 individuals who were approached (75.1%) completed the general questionnaire [47]. Of these, 408 respondents (63.7%) reported having one or more household pets and 401 (98%) completed the supplemental pet-specific questionnaire. Ownership of dogs, cats, exotic companion animals (such as gerbils and rabbits), reptiles and birds were reported. In addition to having household pets, 16 (4.0%) of the respondents reported they lived on a farm where livestock, horses, or poultry were kept.

### Dog-owning households

Dogs were the most frequently reported owned species, with 68.3% [(274/401); 95% CI: 63.5, 72.9] of respondents reporting one or more household dogs. For those respondents listing the number of dogs in the household (n=254), there was a mean (SD) of 1.3 (0.6) dogs per household (range: 1–6). The location of the dog was provided by 239 households; 6 households (2.5 %) had 1 or more outdoor only dogs, 231 (96.7 %) had one or more indoor/outdoor dogs, and 2 (0.8%) had both outdoor only and indoor/outdoor dogs.

Household demographics, animal husbandry, attachment and infection control practices for dog-owning households are listed in Table 2. The majority (52%) of households surveyed had one or more higher risk individuals. Forty percent of the dog-owning respondents recalled having ever received information about pet-associated zoonotic diseases or precautions to reduce the risk of disease.

**Table 2:** **Demographics**, **animal husbandry**, **attachment and practices of dog and cat**-**owning households** (**Ontario**, **Canada**)

**Variable**	**Dog (N=274)**		**Cat (N=191)**	
	**N**_**R**_	**N (%)**	**N**_**R**_	**N (%)**
Child under 16 yrs living in household	274	122 (44.5)	191	78 (40.8)
Child under 5 yrs living in household	274	44 (16.1)	191	26 (13.6)
All children living in household under 5 yrs	274	25 (9.1)	191	19 (10.0)
All children living in household 5-16 yrs	274	78 (28.5)	191	52 (27.2)
Adult ≥ 65 yrs living in household	274	35 (12.8)	191	28 (14.7)
Individual living in household ever diagnosed	227	60 (26.4)	162	55 (34.0)
with an immunocompromising condition				
Individual < 5 yrs, ≥ 65 yrs, or ever diagnosed	237	122 (51.5)	169	90 (53.3)
with an immunocompromising condition				
Previous receipt of pet-associated zoonotic	255	102 (40.0)	172	75 (43.6)
disease education^1^				
Any ever bitten anyone	269	29 (10.8)	184	59 (32.1)
Any ever scratched anyone	NA	NA	180	113 (62.8)
Any declawed	NA	NA	188	110 (58.5)
All on flea prevention program^2^	263	200 (76.1)	177	98 (55.4)
All on intestinal parasite prevention program^3^	258	205 (79.5)	162	68 (42.0)
Any drink from toilet bowl	267	41 (15.4)	186	36 (19.4)
Any fed in kitchen	269	176 (65.4)	189	107 (56.6)
Any used for breeding	268	15 (5.6)	183	0
Any used for hunting	269	3 (1.1)	NA	NA
All visit veterinarian at least annually	259	237 (91.5)	184	121 (65.8)
Any inappropriately urinate or defecate in	268		188	
house^4^				
Always		1 (0.4)		7 (3.7)
Often		14 (5.2)		13 (6.9)
Sometimes		74 (27.6)		43 (22.9)
Never		179 (66.8)		125 (66.5)
Litter box(es) cleaned	NA		184	
Daily		NA		73 (39.7)
Every several days		NA		72 (39.1)
Weekly		NA		30 (16.3)
Every 2 weeks or more		NA		9 (4.9)
In the past 3 months, have any been	274		NA	
On leash at a dog park		53 (19.3)		NA
Off leash at a dog park		51 (18.6)		NA
Doggie daycare or boarding		33 (12.0)		NA
		2 (0.7)		NA
When outdoors, how often in a fenced yard or	267		NA	
on leash				
Always		195 (73.0)		NA
Sometimes		58 (21.7)		NA
Never		14 (5.2)		NA
Fed to any of the pets	264		186	
Commercial canned/dry food		244 (92.4)		185 (99.5)
Raw eggs		9 (3.4)		1 (0.5)
Home cooked pet food		15 (5.7)		3 (1.6)
Raw animal product treats		59 (22.4)		0
Home cooked human food		112 (42.4)		17 (9.1)
Commercial processed pet treats		156 (59.1)		70 (37.6)
Raw meat		11 (4.2)		5 (2.7)
Household’s emotional attachment to pet	268		186	
Very attached		247 (92.2)		150 (80.7)
Somewhat attached		21 (7.8)		32 (17.2)
Not very attached		0		2 (1.1)
Not at all attached		0		2 (1.1)
Any children have access to the litter box(es)	NA	NA	74	49 (66.2)
Any children ever clean-up feces^5^	119	29 (24.4)	77	19 (24.7)
Wear gloves (or use scooper for dogs) when cleaning up feces	28	24 (85.7)	18	5 (27.8)
Wash hands after cleaning up feces	26	25 (96.2)	19	19 (100)
Play in area where the pet goes to the bathroom	114	59 (51.8)	NA	
If play in area, feces removed	59		NA	
Immediately		12 (20.3)		NA
Daily		13 (22.0)		NA
Weekly		24 (40.7)		NA
Greater than weekly		10 (17.0)		NA
Any children touch the pet	118	116 (98.3)	77	72 (93.5)
Children wash hands after touching the pet	115		70	
Always		5 (4.4)		2 (2.9)
Usually		31 (27.0)		25 (35.7)
Sometimes		53 (46.1)		26 (37.1)
Never		26 (22.6)		17 (24.3)
Sleeps in one of the children’s beds^6^	115		71	
Always		9 (7.8)		11 (15.5)
Usually		6 (5.2)		10 (14.1)
Sometimes		15 (13.0)		21 (29.6)
Never		85 (73.9)		29 (40.9)
Licks one of the children’s faces^7^	115		NA	
Daily		7 (6.1)	NA	
Often		21 (18.3)		NA
Sometimes		50 (43.5)		NA
Never		37 (32.2)		NA
Children’s emotional attachment to the pet	114		77	
Very attached		82 (71.9)		52 (67.5)
Somewhat attached		25 (21.9)		14 (18.2)
Not very attached		5 (4.4)		5 (6.5)
Not at all attached		2 (1.8)		6 (7.8)

^1^Respondent recalled having ever received information from any source about diseases that they can get from pets or precautions to take with pets to reduce the risk of disease.

^2^If respondent reported they did not know, they were excluded from analysis: dogs (n=5); cats (n=9).

^3^If respondent reported they did not know, they were excluded from analysis: dogs (n=11); cats (n=20).

^4^Cats: Frequency of urination or defection outside of the litter box in the house; dogs: as written. Dogs/cats: Often (at least once per week), sometimes (less than once per week).

^5^Cats: Children ever clean the litter box(es); dogs: as written.

^6^Usually (3–6 nights per week), sometimes (less than 3 nights per week).

^7^Often (several times per week).

N_R_: Number responding to question. Blank and “don’t know” responses were excluded.

NA: Not asked.

Most (76-80%) dog-owning households had all dogs on flea or endoparasite prevention programs, with 92% of respondents taking all dogs to a veterinarian at least annually. High risk feeding or drinking habits were observed in many households. Most households (65%) fed dogs in the kitchen and some (15%) reported dogs drank from the toilet. High-risk foods were fed to dogs in 28% of households with raw animal product treats (e.g. pig ears) fed most frequently (22%), followed by raw meat (4%), and raw eggs (3%).

Potential contact between household children (< 16 yrs) and fecal material was frequently reported, through their involvement in cleaning up fecal material (24%) or playing in the same area where the dog tended to defecate (52%; 58% of these respondents stated feces were removed weekly or less often). Close contact between dogs and children was often reported, as respondents stated the dog slept in a child’s bed (26%) or licked a child’s face (68%) sometimes or more frequently. Hand washing was reported after children picked up feces (96%), and sometimes or greater following contact with the dog (77%).

In most cases the presence of higher risk individuals in the household or recalled receipt of zoonotic disease education were not significantly associated with the use of different or additional husbandry or infection control practices compared to other households (Table 3). Households with all children less than 5 yrs of age were less likely to report children cleaned up the dog’s feces (OR: 0.05; 95% CI: 0–0.3), but more likely to report a dog ever licked a child’s face (OR: 4.1; 1.1-23.5), than households with all children ≥ 5 yrs.

**Table 3:** **Significant univariable associations between outcomes related to animal husbandry and infection control practices and risk factors reported by pet**-**owning households** (**Ontario**, **Canada**)

						
**Outcome (species)**^**1**^	**All children < 5 yrs (Exposed; N)**^**2**^	**All children ≥ 5 yrs (Unexposed; N)**^**2**^	**Risk Exposed**	**Risk Unexposed**	**Odds Ratio**^**3**^	**95% CI**^**3**^	***P*-value**^**3**^
Any children have access to the litter boxes (cat)							
	4	40	25.0%	78.4%	0.1	0.02-0.4	0.0004
Any children clean up feces (cat)							
	0	18	0%	35.3%	0.07*	0-0.5	0.002
Any children touch pet (cat)							
	13	52	72.2%	100%	0.04*	0-0.3	0.001
Never sleep in children’s bed (cat)							
	10	14	83.3%	26.9%	12.9	2.4-135.9	0.001
Any children clean-up feces (dog)							
	0	28	0%	36.8%	0.05*	0-0.3	0.0002
Ever lick children’s face (dog)							
	20	45	87.0%	61.6%	4.1	1.1-23.5	0.04
**Outcome (species)**^**1**^	**Higher Risk Member (Exposed; N)**^**4**^	**No Higher Risk Member (Unexposed; N)**^**4**^	**Risk Exposed**	**Risk Unexposed**	**Odds Ratio**^**3**^	**95% CI**^**3**^	***P*-value**^**3**^
Within past 3 months, any off leash at a dog park (dog)							
	15	29	12.3%	25.2%	0.4	0.2-0.9	0.02
Household very attached (dog)							
	106	109	88.3%	97.3%	0.2	0.04-0.8	0.01
Dump aquarium water in bathtub, shower, kitchen sink, or bathroom sink (fish)							
	6	12	25.0%	60.0%	0.2	0.05-0.9	0.04
Household very attached (bird)							
	6	0	54.6%	0%	11.5*	1.3-Inf	0.02

^1^Univariable exact logistic regression used to model the likelihood of each husbandry and infection control outcome as a function of selected risk factors (all children < 5 yrs; higher risk member in household). Each row represents a separate regression model.

^2^Performed for children-specific outcomes. Comparison between households with all children < 5 yrs (exposed) and households with all children ≥ 5 yrs (unexposed).

^3^Odds ratio, 95% confidence interval of the odds ratio (95% CI), and *P*-value computed using exact logistic regression.

^4^Comparision between households with ≥ 1 higher risk members [i.e., < 5 yrs, ≥ 65 yrs, ever diagnosed with an immunocompromising condition (exposed)] and households without higher risk members (unexposed).

*Median unbiased estimate computed since odds ratio calculated to be infinite.

### Cat-owning households

One hundred ninety one respondents (47.6%; 95% CI: 42.7, 52.6) reported owning one or more household cats. For those respondents listing the number of cats in the household (n=178), there was a mean (SD) of 1.7 (1.4) cats per household (range: 1–11). The location of the cat was provided by 176 households; 7 households (4%) had one or more outdoor only cats, 114 (65%) had one or more indoor only cats, and 73 (41%) had one or more indoor/outdoor cats. However, 41 (56%) of households with indoor/outdoor cats kept cats restrained on a leash when outdoors.

Household demographics, animal husbandry, attachment and infection control practices for cat-owning households are listed in Table 2. The majority (53%) of households had one or more higher risk individuals. Forty-four percent of the cat-owning respondents recalled having ever received information about pet-associated zoonotic diseases or precautions to reduce the risk of disease.

Bites and scratches ever occurring by any of the current household cats were frequently reported (32% and 63%, respectively). Approximately half of responding households had all cats on flea (55%) or endoparasite (42%) prevention programs, with 66% of households taking all cats to a veterinarian at least annually. High risk feeding and drinking habits were frequently observed, with many households (57%) having fed cats in the kitchen. High-risk foods were fed to cats in 3% of households with raw meat (3%) and raw eggs (0.5%) reported.

Potential contact between children and fecal material was frequently reported, through their involvement in cleaning up fecal material (25%) or access to the litter box (66%). Twenty-one percent of respondents reported the litter box was cleaned weekly or less often; this percentage was similar for households with and without children. Close contact between cats and children was frequently reported, as 59% of households reported a cat slept in a child’s bed. Hand washing was reported after children picked up feces (100%) and sometimes or greater following contact with the cat (76%).

The presence of only children under 5 yrs of age in the household was significantly associated with the occurrence of several infection control practices compared to households with all children 5 yrs of age or older (Table 3). Households with all children less than 5 yrs were less likely to report children cleaned up the cat’s feces (OR: 0.07; 95% CI: 0–0.5), less likely to touch the cat (OR: 0.04; 95% CI: 0–0.3), less likely to have access to the litter box (OR: 0.1; 95% CI: 0.02-0.4), and more likely for the cat to never sleep in the child’s bed (OR: 12.9; 95% CI: 2.4-135.9), than households in which all children were ≥ 5 yrs.

### Fish-owning households

Fifty-three respondents (13.2%; 95% CI: 10.1, 16.9) reported one or more household fish (median 4 fish per household; range: 1–60). Household demographics, animal husbandry, attachment and infection control practices are listed in Table 4. The majority (53%) of households had one or more higher risk individuals. Thirty percent of the fish-owning respondents recalled having ever received information about pet-associated zoonotic diseases or precautions to reduce the risk of disease.

**Table 4:** **Demographics**, **animal husbandry**, **attachment and practices of fish and bird**-**owning households** (**Ontario**, **Canada**)

			
**Variable**	**Fish (N=53)**		**Bird (N=22)**	
	**N**_**R**_	**N (%)**	**N**_**R**_	**N (%)**
**Household Demographics**				
Child under 16 yrs living in household	53	34 (64.2)	22	8 (36.4)
Child under 5 yrs living in household	53	10 (18.9)	22	1 (4.6)
Adult ≥ 65 yrs living in household	53	2 (3.8)	22	4 (18.2)
Individual living in household ever diagnosed	42	15 (35.7)	20	7 (35.0)
with an immunocompromising condition				
Individual < 5 yrs, ≥ 65 yrs, or ever diagnosed	45	24 (53.3)	21	12 (57.1)
with an immunocompromising condition				
Previous receipt of pet-associated zoonotic disease	47	14 (29.8)	21	7 (33.3)
education^1^				
Housing cleaned^2^	52		21	
Daily		1 (1.9)		4 (19.1)
Every several days		1 (1.9)		3 (14.3)
Weekly		17 (32.7)		8 (38.1)
Every 2 weeks		12 (23.1)		3 (14.3)
Greater than every 2 weeks		21 (40.4)		3 (14.3)
Dump aquarium water	50		NA	
Toilet		20 (40.0)		NA
Bathtub or shower		3 (6.0)		NA
Kitchen sink		12 (24.0)		NA
Bathroom sink		8 (16.0)		NA
Outside		19 (38.0)		NA
Other		3 (6.0)		NA
Household’s emotional attachment to pet	50		21	
Very attached		5 (10.0)		6 (28.6)
Somewhat attached		22 (44.0)		10 (47.6)
Not very attached		19 (38.0)		5 (23.8)
Not at all attached		4 (8.0)		0
Any children ever clean the animal’s housing^2^	33	10 (30.3)	8	3 (37.5)
Wear gloves when cleaning	10	0	3	1 (33.3)
Wash hands after cleaning	10	9 (90.0)	3	2 (66.6)
Children touch the pet (or water for fish)	30	12 (40.0)	8	4 (50.0)
Children wash hands after touching	12		4	
Always		4 (33.3)		2 (50.0)
Usually		4 (33.3)		1 (25.0)
Sometimes		3 (25.0)		0
Never		1 (8.3)		1 (25.0)
Children’s emotional attachment to the pet	30		8	
Very attached		3 (10.0)		1 (12.5)
Somewhat attached		14 (46.7)		6 (75.0)
Not very attached		12 (40.0)		1 (12.5)
Not at all attached		1 (3.3)		0

^1^Respondent recalled having ever received information from any source about diseases that they can get from pets or precautions to take with pets to reduce the risk of disease.

^2^“Housing” refers to aquaria (fish) and cages (birds).

N_R_: Number responding to question. Blank and “don’t know” responses were excluded.

NA: Not asked.

Forty-four percent of respondents stated they disposed of used aquarium water by pouring it into a location with direct human or food contact [i.e., kitchen sink (24%), bathroom sink (16%), bathtub/shower (6%)]. Those households with higher risk members were less likely to dispose of aquarium water in this manner than households without these members (OR: 0.2; 95% CI: 0.05-0.9; Table 3). When children were present in a household, they were frequently involved with cleaning the aquarium (30%) and touching the fish or water (40%). Over 90% of households reported that children washed their hands after cleaning, or washed their hands sometimes or greater after touching the fish or water. A large percentage (43-46%) of households reported the household and children (if present) were not very, or not at all, attached to the fish.

### Bird-owning households

Twenty-two respondents (5.5%; 95% CI: 3.5, 8.2) reported one or more household birds (median one bird per household; range: 1–40). Household demographics, animal husbandry, attachment and infection control practices for bird-owning households are listed in Table 4. The majority (57%) of households had one or more higher risk individuals. Thirty-three percent of the bird-owning respondents recalled having ever received information about pet-associated zoonotic diseases or precautions to reduce the risk of disease. Households with higher risk individuals were more likely to be very attached to the birds than households without these individuals (OR: 11.5; 95% CI: 1.3-infinite; Table 3).

### Exotic companion mammal-owning households

Twenty-nine respondents (7.2%; 95% CI: 4.9, 10.2) reported one or more household exotic mammals (median 1 per household; range: 1–13), consisting of hamsters (n=10), rabbits (6), guinea pigs (5), rats/mice (4) and hedgehogs, chinchillas and gerbils (2 each). Household demographics, animal husbandry, attachment and infection control practices for exotic mammal-owning households are listed in Table 5. Many (46%) households had one or more higher risk individuals. Three households (10%) had at least one child less than 5 yrs of age. Forty-one percent of the respondents recalled having ever received information about pet-associated zoonotic diseases or precautions to reduce the risk of disease.

**Table 5:** **Demographics**, **animal husbandry**, **attachment and practices of exotic companion mammal and reptile**-**owning households (Ontario**, **Canada)**

			
**Variable**	**Exotic Companion Mammal**^**1 **^**(N=29)**		**Reptile (N=22)**	
	**N**_**R**_	**N (%)**	**N**_**R**_	**N (%)**
**Household Demographics**				
Child under 16 yrs living in household	29	19 (65.5)	22	10 (45.5)
Child under 5 yrs living in household	29	3 (10.3)	22	2 (9.1)
Adult ≥ 65 yrs living in household	29	2 (6.9)	22	0
Individual living in household ever diagnosed	23	8 (34.8)	19	9 (47.4)
with an immunocompromising condition				
Individual < 5 yrs, ≥ 65 yrs, or ever diagnosed	26	12 (46.2)	20	11 (55.0)
with an immunocompromising condition				
Previous receipt of pet-associated zoonotic	27	11 (40.7)	17	7 (41.2)
disease education^2^				
**Animal Husbandry, Attachment and Practices**				
Cage cleaned	29		21	
Daily		2 (6.9)		1 (4.8)
Every several days		6 (20.7)		3 (14.3)
Weekly		18 (62.1)		6 (28.6)
Every 2 weeks		3 (10.3)		5 (23.8)
Greater than every 2 weeks		0		6 (28.6)
Animals allowed to roam through house	NA	NA	22	2 (9.1)
Animals allowed to roam in kitchen	NA	NA	22	2 (9.1)
Animals/cages washed in kitchen sink	NA	NA	22	3 (13.6)
Animals/cages washed in bathtub or bathroom	NA	NA	18	5 (27.8)
sink				
Household’s emotional attachment to pet	29		21	
Very attached		16 (55.2)		3 (14.3)
Somewhat attached		11 (37.9)		11 (52.4)
Not very attached		2 (6.9)		4 (19.1)
Not at all attached		0		3 (14.3)
**Questions answered by households with one or more children under 16 yrs**				
Any children ever clean the animal’s cage	19	12 (63.2)	10	4 (40.0)
Wear gloves when cleaning	12	1 (8.3)	3	0
Wash hands after cleaning	12	11 (91.7)	4	3 (75.0)
Animals roam in children’s room	NA	NA	8	0
Children touch the pet	19	19 (100)	9	7 (77.8)
Children wash hands after touching	19		7	
Always		8 (42.1)		4 (57.1)
Usually		8 (42.1)		0
Sometimes		3 (15.8)		2 (28.6)
Never		0		1 (14.3)
Children’s emotional attachment to the pet	18		10	
Very attached		11 (61.1)		1 (10.0)
Somewhat attached		6 (33.3)		5 (50.0)
Not very attached		1 (5.6)		3 (30.0)
Not at all attached		0		1 (10.0)

^1^Exotic companion mammals included rabbits, hedgehogs, and rodents (such as gerbils, hamsters, guinea pigs, chinchillas, mice, and rats).

^2^Respondent recalled having ever received information from any source about diseases that they can get from pets or precautions to take with pets to reduce the risk of disease.

N_R_: Number responding to question. Blank and “don’t know” responses were excluded.

NA: Not asked.

When children were present in a household, they were frequently involved with cleaning the animal’s cage (63%) or touching the pet (100%). Most children reportedly washed their hands after cleaning the cage (92%) or washed their hands sometimes or greater after touching the pet (100%). A minority (42%) always washed their hands after touching the pet. Most respondents (> 90%) reported the household and children (if present) were very or somewhat attached to the pets. No statistically significant associations between husbandry/infection control practices and risk factors were detected.

### Reptile-owning households

Twenty-two respondents (5.5%; 95% CI: 3.5, 8.2) reported one or more household reptiles (median one per household; range: 1–11), consisting of lizards (n=9), snakes (5), and turtles (9). Household demographics, animal husbandry, attachment and infection control practices for reptile-owning households are listed in Table 5. Many households (55%) had one or more higher risk individuals, with a high proportion (47%) having been diagnosed with an immunocompromising condition. Two households (9%) had at least one child less than 5 yrs of age. Forty-one percent of the reptile-owning respondents recalled having ever received information about pet-associated zoonotic diseases or precautions to reduce the risk of disease.

Three respondents (14%) reported the pet reptile was allowed to roam through the house, kitchen or washed in the kitchen sink. When children were present in a household, they were often involved with cleaning the animal’s cage (40%) or touching the pet (78%). In one household in which children touched the reptile, all children (n=2) were less than 5 yrs. Most respondents reported the children washed their hands after cleaning the cage (75%) or washed their hands sometimes or greater after touching the pet (86%). However, only 57% always washed their hands after touching the reptile. Many respondents reported the household and children (if present) were not very, or not at all, attached to the reptile (33% and 40%, respectively). No statistically significant associations between husbandry/infection control practices and risk factors were detected.

### Farm-owning households

Sixteen respondents (4.0% of those with household pets; 95% CI: 2.3, 6.4) reported they lived on a farm. Respondents most commonly listed horses (n=9), chickens (5), cattle and pigs (3 each) as the farm animals present. Fifty percent (7/14) of households for which the data were provided had one or more higher risk individuals. When children were present [6/16 (38%)], they were often involved in higher risk activities. Most [5/6 (83%)] of such households reported children helped feed and clean-up after the farm animals at least sometimes, and in most households [5/6 (83%)] children usually or always washed their hands after contact with the animals or their housing.

## Discussion

As pet-human contact frequently occurs and disease transmission is overall rarely reported [47], in most situations such contact should not pose a substantial health risk. However, there are practices that may increase pathogen exposure and individuals (< 5yrs, ≥ 65yrs, or immunocompromised) for whom these risks are known or presumed to be greater. Reported husbandry and infection control practices in this study indicated opportunities for exposure to pathogens. Respondents reported household children had close contact with pets (e.g., face licking, sleeping in beds, cleaning up pet fecal material). These findings are similar to a previous study in which dogs licked their owners’ face (50%) and dogs and cats slept in an adult’s bed (18%, 30%, respectively) [37]. The oral cavity of the dog and cat contain numerous potentially zoonotic opportunistic pathogens [49] and face licking can result in transmission of bacteria such as *Pasteurella* spp. and *Capnocytophaga canimorsus*[50], potentially resulting in life-threatening disease [22]. For these reasons, some discourage young children and immunocompromised individuals from regularly allowing face licking by pets [50]. The disease risk associated with dogs and cats sleeping in a child’s bed is largely unquantified, however sleeping with pets has been identified as a risk factor for several diseases, prompting some to discourage this practice by higher risk individuals [50].

Routine preventive veterinary care and husbandry practices are important techniques for reducing zoonotic disease transmission between pets and people [30,35]. Consistent with previous studies [40,44], most cat- and dog-owning households had these animals examined at least annually by a veterinarian, which allows for identification and education of potential disease concerns and facilitates implementation of an appropriate preventive medicine program. The differences observed between dog and cat preventative veterinary care, with cats having lesser veterinary contact, has been previously noted [40,44]. Since cats have less contact with the veterinary healthcare system yet have close interactions with household members, educational approaches that reach beyond the veterinary clinic may be required.

Concerning husbandry practices were observed in all owned species. Feeding of raw meat or egg products are well-established risk factors for salmonellosis in dogs [45,51] and presumably cats. Raw animal product treats (i.e. pig ears) have historically been a concern for *Salmonella* infections in pets and pet-owners, with outbreaks identified [52,53]. Although feeding these items is discouraged, particularly in higher risk households [54], many households reported this practice. These risks are further compounded by feeding pets in the kitchen (frequently reported in this study) as documented in an outbreak investigation of salmonellosis in infants linked to *Salmonella*-contaminated pet food [55]. As several outbreaks of human salmonellosis have been associated with contaminated dry commercial pet food [55,56], feeding pets in the kitchen should be discouraged, especially in higher risk households [29]. Concerning husbandry practices were frequently reported in reptile-owning homes (e.g., infrequent hand washing, permitting them to roam freely through a home or kitchen). Due to the high prevalence of *Salmonella* shedding by healthy reptiles [57] and the high incidence of human salmonellosis attributed to reptile contact [24], the reported practices are strongly discouraged [34,58].

Hand hygiene serves a critical role in reducing the risk of zoonotic infections. Our results were similar to previous studies in which 96% of dog owners reported they usually or greater washed their hands after picking up feces [36] and 15% always washed their hands after touching the dog [37]. For lower risk circumstances, such as contact with a dog or cat, the level of hand hygiene documented in our study is likely adequate; however, in higher-risk situations, such as following contact with a reptile, hand washing should always occur.

Across all animal species that were owned, many households had an individual at higher risk of infections (< 5yrs, ≥ 65yrs, immunocompromised). Although pet ownership has previously been commonly reported in households with individuals who are immunocompromised [15,38,39] and of extremes of age [40-43], it was concerning to observe this trend with higher risk species such as reptiles, which are discouraged from being kept in higher risk households [29,31-34,58]. However, this finding was not surprising as a previous study involving this respondent group found households with higher risk individuals did not differ from the remaining households regarding their knowledge or perceived risk of pet-associated disease or recall of having received information regarding pet-associated disease risks [47]. Physicians and veterinarians are in an important position to offer guidance on proper pet selection. In order to tailor recommendations, physicians should obtain a history of contact with pets or other animals as part of every wellness evaluation, especially for individuals at higher risk of zoonotic disease, and veterinarians should utilize methods to encourage clients to divulge health information relevant to disease risk status for household members.

Despite evaluating over 50 practices consistent with existing recommendations for individuals at greater risk for infectious disease [29-35], few significant associations were identified. Most of those identified suggested a lower risk of practice occurrence in households with young children; the extent to which such decisions were purposefully implemented versus simply a consequence of young age is unknown. The finding that young children were at increased risk to be licked on the face by the household dog was expected due to their age and size, but nonetheless concerning because of reports of lick-associated infections in children [22,50].

The limited use of husbandry and infection control practices by households with higher risk individuals was disappointing but not surprising. Previous research on this respondent group found pet owners were overall unconcerned about pet-associated zoonoses and a minority (36%) recalled previously receiving zoonotic disease education [47]. Most respondents were comfortable with their level of knowledge of, and methods to reduce, zoonotic disease risk [47]. While it is unclear whether participants had reasonable knowledge of those areas, their perceived competency could reduce the impetus to seek out further information.

General zoonotic disease counselling was previously associated with statistically higher zoonotic disease knowledge scores in this respondent group [47], yet no such difference was observed in husbandry or infection control practices in the present study. This discrepancy may have arisen due to the minimally defined nature of this variable (i.e. timing and quality of information provided were not assessed), but it is also possible that although education imparted improved knowledge of zoonotic disease risks, due to a lack of concern for pet-associated zoonoses or competing time commitments, infection control and husbandry practices were unaffected. It is also possible that education regarding diseases was not accompanied by adequate information about preventive measures.

This study identified numerous pet husbandry and infection control practices that may increase disease risk in pet-owning households. In order to determine the relative importance of these practices and educational efforts that should be employed, targeted observational studies are needed. While guidelines for infection-control practices in higher risk households are available, it appears that individuals in such households are no better educated on the topic of pet-associated zoonoses [47], nor do their husbandry or infection control practices differ from other households. Educational efforts are needed for households with individuals at higher risk of infections. Given the multifaceted nature of this topic, with communication barriers among physicians, veterinarians and the public [59-61], all members of the family healthcare team (i.e., physicians, veterinarians, public health) must work together to reach the common goal of reducing the public’s pet-associated disease risks.

Several biases may have been introduced into this study and affected the results. We acquired our data from a convenience sample from the waiting room of general practice physician offices from a limited geographic area. All Canadian residents receive medically necessary healthcare services at no charge [62], reducing the potential that variable access to physicians would result in a biased study population. As previously discussed [47], based on census data, our sample appeared to be representative of the region. Response bias cannot be excluded, however as blank and “don’t know” responses were uncommon (<20% for each question with the majority <10%), the magnitude of this bias is likely limited. As many questions addressed health-related actions taken by respondents for which they may have known what answers were ideal, respondents may have misclassified household practices. In these cases, information provided would suggest better husbandry and infection control practices than actually existed. This was perhaps most likely for questions surrounding hand washing [63]. As only a single individual from each household participated in the study, it is possible reported observations and perceptions may not have been representative of the household. Due to the nature of this study, small sample sizes were often encountered, which in some cases prohibited analysis and in others may have limited statistical power. Furthermore, small sample sizes prohibited multivariable analysis and the ability to investigate confounding. A large number of individual regression analyses were conducted and corrections were not made for this multiplicity of testing; estimates are likely to have an increased type I error and fewer significant associations may exist than reported.

## Conclusions

This study characterized pet husbandry and infection control practices that relate to zoonotic disease risks in pet-owning households in Ontario, Canada, and identified deficiencies that could result in exposure to pathogens and human zoonotic infections. Individuals at higher risk of infections were frequently present in pet-owning households, regardless of species. With few exceptions, households with members at higher risk for infectious disease and those who recalled having received education on pet-associated disease risks followed similar practices to households without these individuals or education. Targeted educational efforts are indicated for households with individuals at higher risk of infections and those with high-risk species (e.g., reptiles). Healthcare team members, including veterinarians, physicians and public health practitioners, have the potential to serve as valuable resources in reducing pet-associated disease risks, however all must jointly work to address this area and devote more attention toward the public’s interaction with animals. Further research is needed to determine the reasoning behind household infection control and husbandry practices and the respective roles of education and perceptions in shaping these practices.

## Competing interests

The authors declare they have no competing interests.

## Authors' contributions

All authors participated in the study concept, design and questionnaire development. JWS enrolled participating clinics and administered questionnaires to participating patients. JWS was responsible for data analysis, data interpretation and manuscript preparation. All authors provided input on data analysis, interpretation and final manuscript development. All authors have approved the final version of the manuscript.

## Pre-publication history

The pre-publication history for this paper can be accessed here:

http://www.biomedcentral.com/1471-2458/13/520/prepub

## Supplementary Material

Additional file 1 Supplemental questionnaire for households with pets. Click here for file

## References

[B1] Butler JR Bingham J Demography and dog-human relationships of the dog population in Zimbabwean communal lands Vet Rec 2000 147 16 442 446 10.1136/vr.147.16.442 11079440

[B2] Baldock FC Alexander L More SJ Estimated and predicted changes in the cat population of Australian households from 1979 to 2005 Aust Vet J 2003 81 5 289 292 10.1111/j.1751-0813.2003.tb12577.x 15084040

[B3] Downes M Canty MJ More SJ Demography of the pet dog and cat population on the island of Ireland and human factors influencing pet ownership Prev Vet Med 2009 92 1–2 140 149 19700212 10.1016/j.prevetmed.2009.07.005

[B4] Slater MR Di Nardo A Pediconi O Villa PD Candeloro L Alessandrini B Del Papa S Cat and dog ownership and management patterns in central Italy Prev Vet Med 2008 85 3–4 267 294 18374434 10.1016/j.prevetmed.2008.02.001

[B5] Murray JK Browne WJ Roberts MA Whitmarsh A Gruffydd-Jones TJ Number and ownership profiles of cats and dogs in the UK Vet Rec 2010 166 6 163 168 10.1136/vr.b4712 20139379

[B6] Perrin T The Business Of Urban Animals Survey: the facts and statistics on companion animals in Canada Can Vet J 2009 50 1 48 52 19337613 PMC2603652

[B7] Alves MC Matos MR Reichmann ML Dominguez MH Estimation of the dog and cat population in the State of Sao Paulo Rev Saude Publica 2005 39 6 891 897 16341397 10.1590/s0034-89102005000600004

[B8] American Veterinary Medical Association U.S. pet ownership & demographics sourcebook 2007 Schaumburg (Illinois): American Veterinary Medical Association

[B9] Friedmann E Son H The human–companion animal bond: how humans benefit Vet Clin N Am: Small Anim Pract 2009 39 2 293 326 10.1016/j.cvsm.2008.10.015 19185195

[B10] Reaser JK Clark EE Meyers NM All creatures great and minute: a public policy primer for companion animal zoonoses Zoonoses Public Health 2008 55 8–10 385 401 18399943 10.1111/j.1863-2378.2008.01123.x

[B11] Poresky RH Hendrix C Differential effects of pet presence and pet-bonding on young children Psychol Rep 1990 67 1 51 54 10.2466/pr0.1990.67.1.51 2236419

[B12] Melson GF Schwarz RL Beck AM Importance of companion animals in children's lives-implications for veterinary practice J Am Vet Med Assoc 1997 211 12 1512 1518 9412676

[B13] Carmack BJ The role of companion animals for persons with AIDS/HIV Holist Nurs Pract 1991 5 2 24 31 1984013 10.1097/00004650-199101000-00007

[B14] Castelli P Hart LA Zasloff RL Companion cats and the social support systems of men with AIDS Psychol Rep 2001 89 1 177 187 10.2466/pr0.2001.89.1.177 11729540

[B15] Conti L Lieb S Liberti T Wiley-Bayless M Hepburn K Diaz T Pet ownership among persons with AIDS in three Florida counties Am J Public Health 1995 85 11 1559 1561 10.2105/AJPH.85.11.1559 7485673 PMC1615702

[B16] Raina P Waltner-Toews D Bonnett B Woodward C Abernathy T Influence of companion animals on the physical and psychological health of older people: an analysis of a one-year longitudinal study J Am Geriatr Soc 1999 47 3 323 329 10078895 10.1111/j.1532-5415.1999.tb02996.x

[B17] Hale CR Scallan E Cronquist AB Dunn J Smith K Robinson T Lathrop S Tobin-D'Angelo M Clogher P Estimates of enteric illness attributable to contact with animals and their environments in the United States Clin Infect Dis 2012 54 Suppl 5 S472 S479 10.1093/cid/cis051 22572672

[B18] Weese JS Fulford MB Companion Animal Zoonoses Iowa: Wiley-Blackwell 2011 3 298

[B19] Friedman CR Hoekstra RM Samuel M Marcus R Bender J Shiferaw B Reddy S Ahuja SD Helfrick DL Hardnett F Carter M Anderson B Tauxe RV Emerging Infections Program FoodNet Working Group: **Risk factors for sporadic *****Campylobacter *****infection in the United States**: **a case**–**control study in FoodNet sites** Clin Infect Dis 2004 38 Suppl 3 S285 S296 15095201 10.1086/381598

[B20] Tenkate TD Stafford RJ Risk factors for *Campylobacter* infection in infants and young children: a matched case–control study Epidemiol Infect 2001 127 3 399 404 11811871 10.1017/s0950268801006306 PMC2869763

[B21] Younus M Wilkins MJ Davies HD Rahbar MH Funk J Nguyen C Siddiqi AE Cho S Saeed AM The role of exposures to animals and other risk factors in sporadic, non-typhoidal *Salmonella* infections in Michigan children Zoonoses Public Health 2010 57 7–8 e170 e176 20202185 10.1111/j.1863-2378.2010.01324.x

[B22] Kobayaa H Souki RR Trust S Domachowske JB *Pasteurella multocida* meningitis in newborns after incidental animal exposure Pediatr Infect Dis J 2009 28 10 928 929 10.1097/INF.0b013e3181a81f0f 19730153

[B23] De Boer MG Lambregts PC Van Dam AP Van T Wout JW **Meningitis caused by *****Capnocytophaga canimorsus***: **when to expect the unexpected** Clin Neurol Neurosurg 2007 109 5 393 398 10.1016/j.clineuro.2007.02.010 17408852

[B24] Mermin J Hutwagner L Vugia D Shallow S Daily P Bender J Koehler J Marcus R Angulo FJ Emerging Infections Program FoodNet Working Group: **Reptiles**, **amphibians**, **and human *****Salmonella *****infection**: **a population**-**based**, **case**–**control study** Clin Infect Dis 2004 38 Suppl 3 S253 S261 15095197 10.1086/381594

[B25] Lin AE Davies JE Occurrence of highly fluoroquinolone-resistant and methicillin-resistant *Staphylococcus aureus* in domestic animals Can J Microbiol 2007 53 7 925 929 10.1139/W07-062 17898848

[B26] Platell JL Trott DJ Johnson JR Heisig P Heisig A Clabots CR Johnston B Cobbold RN Prominence of an O75 clonal group (clonal complex 14) among non-ST131 fluoroquinolone-resistant *Escherichia coli* causing extraintestinal infections in humans and dogs in Australia Antimicrob Agents Chemother 2012 56 7 3898 3904 10.1128/AAC.06120-11 22526317 PMC3393427

[B27] Mani I Maguire JH Small animal zoonoses and immunocompromised pet owners Top Companion Anim Med 2009 24 4 164 174 10.1053/j.tcam.2009.07.002 19945084

[B28] Abbas AK Lichtman AH Pillai S Congenital and acquired immunodeficiencies Cellular and molecular immunology 2007 6 Philadelphia: Saunders 463 488

[B29] Angulo FJ Glaser CA Juranek DD Lappin MR Regnery RL Caring for pets of immunocompromised persons Can Vet J 1995 36 4 217 222 17424395 PMC1686928

[B30] Glaser CA Powers EL Greene CE Greene CE Zoonotic infections of medical importance in immunocompromised humans Infectious diseases of the dog and cat 2012 4 St. Louis: Elsevier 1141 1162

[B31] Centers for Disease Control and Prevention **Guidelines for preventing opportunistic infections among hematopoietic stem cell transplant recipients**: **recommendations from CDC**, **Infectious Disease Society of America**, **American Society of Blood and Marrow Transplantation** MMWR Recomm Rep 2000 49 RR-10 1 125 CE1-7 11718124

[B32] Centers for Disease Control and Prevention **Guidelines for the Prevention and Treatment of Opportunistic Infections among HIV**-**exposed and HIV**-**infected children**: **recommendations from CDC**, **the National Institutes of Health**, **the HIV Medicine Association of the Infectious Diseases Society of America**, **the Pediatric Infectious Diseases Society**, **and the American Academy of Pediatrics** MMWR Recomm Rep 2009 58 RR-11 1 166 19730409 PMC2821196

[B33] Pickering LK Marano N Bocchini JA Angulo FJ Committee on Infectious Diseases: **Exposure to nontraditional pets at home and to animals in public settings**: **risks to children** Pediatrics 2008 122 4 876 886 10.1542/peds.2008-1942 18829816

[B34] Centers for Disease Control and Prevention Compendium of Measures to Prevent Disease Associated with Animals in Public Settings, 2011 MMWR 2011 60 RR-4 1 24 21546893

[B35] Lappin MR Pet ownership by immunocompromised people Compend Contin Educ Pract Vet 2002 24 5A 16 25

[B36] Westgarth C Pinchbeck GL Bradshaw JW Dawson S Gaskell RM Christley RM Dog-human and dog-dog interactions of 260 dog-owning households in a community in Cheshire Vet Rec 2008 162 14 436 442 10.1136/vr.162.14.436 18390853

[B37] Overgaauw PA Van Zutphen L Hoek D Yaya FO Roelfsema J Pinelli E Van Knapen F Kortbeek LM Zoonotic parasites in fecal samples and fur from dogs and cats in The Netherlands Vet Parasitol 2009 163 1–2 115 122 19398275 10.1016/j.vetpar.2009.03.044

[B38] Siegel JM Angulo FJ Detels R Wesch J Mullen A AIDS diagnosis and depression in the Multicenter AIDS Cohort Study: the ameliorating impact of pet ownership AIDS Care 1999 11 2 157 170 10.1080/09540129948054 10474619

[B39] Abarca VK Del Lopez PJ Pena DA Lopez GJC Pet ownership and health status of pets from immunocompromised children, with emphasis in zoonotic diseases Rev Chilena Infectol 2011 28 3 205 210 10.4067/S0716-10182011000300001 21879144

[B40] Leslie BE Meek AH Kawash GF McKeown DB An epidemiological investigation of pet ownership in Ontario Can Vet J 1994 35 4 218 222 8076276 PMC1686751

[B41] Westgarth C Pinchbeck GL Bradshaw JW Dawson S Gaskell RM Christley RM Factors associated with dog ownership and contact with dogs in a UK community BMC Vet Res 2007 3 5 10.1186/1746-6148-3-5 17407583 PMC1852100

[B42] Westgarth C Heron J Ness AR Bundred P Gaskell RM Coyne KP German AJ McCune S Dawson S Family pet ownership during childhood: findings from a UK birth cohort and implications for public health research Int J Environ Res Public Health 2010 7 10 3704 3729 10.3390/ijerph7103704 21139856 PMC2996187

[B43] Ramon ME Slater MR Ward MP Companion animal knowledge, attachment and pet cat care and their associations with household demographics for residents of a rural Texas town Prev Vet Med 2010 94 3–4 251 263 20122744 10.1016/j.prevetmed.2010.01.008

[B44] Volk JO Felsted KE Thomas JG Siren CW Executive summary of the Bayer veterinary care usage study J Am Vet Med Assoc 2011 238 10 1275 1282 10.2460/javma.238.10.1275 21568772

[B45] Leonard EK Pearl DL Finley RL Janecko N Peregrine AS Reid-Smith RJ Weese JS Evaluation of pet-related management factors and the risk of *Salmonella* spp. carriage in pet dogs from volunteer households in Ontario (2005–2006) Zoonoses Public Health 2011 58 2 140 149 10.1111/j.1863-2378.2009.01320.x 20163574

[B46] Waterloo, Ontario 2006: **Community Profiles** http://www12.statcan.ca/census-recensement/2006/dp-pd/prof/92-591/index.cfm?Lang=E

[B47] Stull JW Peregrine AS Sargeant JM Weese JS Household knowledge, attitudes and practices related to pet contact and associated zoonoses in Ontario Canada. BMC Public Health 2012 12 1 553 10.1186/1471-2458-12-553 PMC3489606 22831165

[B48] Mehta CR Patel NR Exact logistic regression: theory and examples Statist Med 1995 14 19 2143 2160 10.1002/sim.4780141908 8552893

[B49] Forsblom B Sarkiala-Kessel E Kanervo A Vaisanen ML Helander M Jousimies-Somer H Characterisation of aerobic gram-negative bacteria from subgingival sites of dogs–potential bite wound pathogens J Med Microbiol 2002 51 3 207 220 11871615 10.1099/0022-1317-51-3-207

[B50] Chomel BB Sun B Zoonoses in the bedroom Emerg Infect Dis 2011 17 2 167 172 10.3201/eid1702.101070 21291584 PMC3298380

[B51] Kukanich KS Update on *Salmonella* spp. contamination of pet food, treats, and nutritional products and safe feeding recommendations J Am Vet Med Assoc 2011 238 11 1430 1434 10.2460/javma.238.11.1430 21627504

[B52] Clark C Cunningham J Ahmed R Woodward D Fonseca K Isaacs S Ellis A Anand C Ziebell K Muckle A Sockett P Rodgers F Characterization of *Salmonella* associated with pig ear dog treats in Canada J Clin Microbiol 2001 39 11 3962 3968 10.1128/JCM.39.11.3962-3968.2001 11682515 PMC88472

[B53] Centers for Disease Control and Prevention (CDC): **Human salmonellosis associated with animal**-**derived pet treats**-**United States and Canada**, **2005** MMWR Morb Mortal Wkly Rep 2006 55 25 702 705 16810148

[B54] Finley R Reid-Smith R Ribble C Popa M Vandermeer M Aramini J The occurrence and anti-microbial susceptibility of Salmonellae isolated from commercially available pig ear pet treats Zoonoses Public Health 2008 55 8–10 455 461 18631234 10.1111/j.1863-2378.2008.01144.x

[B55] Behravesh CB Ferraro A Deasy M Dato V Moll M Sandt C Rea NK Rickert R Marriott C Warren K Urdaneta V Salehi E Villamil E Ayers T Hoekstra RM Austin JL Ostroff S Williams IT *Salmonella* Schwarzengrund Outbreak Investigation Team: **Human *****Salmonella *****infections linked to contaminated dry dog and cat food**, **2006**–**2008** Pediatrics 2010 126 3 477 483 10.1542/peds.2009-3273 20696725

[B56] Centers for Disease Control and Prevention (CDC) **Notes from the field**: **human *****Salmonella infantis *****infections linked to dry dog food** - **United States and Canada**, **2012** MMWR Morb Mortal Wkly Rep 2012 61 436 22695384

[B57] Hydeskov HB Guardabassi L Aalbaek B Olsen KE Nielsen SS Bertelsen MF *Salmonella* prevalence among reptiles in a zoo education setting Zoonoses Public Health 2013 60 4 291 5 10.1111/j.1863-2378.2012.01521.x 22835051

[B58] Centers for Disease Control and Prevention (CDC) **Reptile**-**associated salmonellosis**-**selected states**, **1998**–**2002** MMWR Morb Mortal Wkly Rep 2003 52 49 1206 1209 14668712

[B59] Grant S Olsen CW Preventing zoonotic diseases in immunocompromised persons: the role of physicians and veterinarians Emerg Infect Dis 1999 5 1 159 163 10.3201/eid0501.990121 10081686 PMC2627689

[B60] Von Matthiessen PW Sansone RA Meier BP Gaither GA Shrader J Zoonotic diseases and at-risk patients: a survey of veterinarians and physicians AIDS 2003 17 9 1404 1406 10.1097/00002030-200306130-00021 12799568

[B61] Hill WA Petty GC Erwin PC Souza MJ A survey of Tennessee veterinarian and physician attitudes, knowledge, and practices regarding zoonoses prevention among animal owners with HIV infection or AIDS J Am Vet Med Assoc 2012 240 12 1432 1440 10.2460/javma.240.12.1432 22657926

[B62] Health Canada Canada Health Act Annual Report 2009–2010 2010 Ottawa, Ontario: 978-1-100-16312-3:4

[B63] Moret L Tequi B Lombrail P Should self-assessment methods be used to measure compliance with handwashing recommendations? A study carried out in a French university hospital Am J Infect Control 2004 32 7 384 390 10.1016/j.ajic.2004.02.004 15525912

