# Supplementary material for: Pet husbandry and infection control practices related to zoonotic disease risks in Ontario, Canada
Source: BMC Public Health. 2013 May 29;13:520. doi: 10.1186/1471-2458-13-520 (PMC3668296; doi:10.1186/1471-2458-13-520)
Supplement: Additional file 1 — Supplemental questionnaire for households with pets. [file 1471-2458-13-520-S1.pdf]

Supplemental Questionnaire for Households with Pets

(If you do not currently have pets in your household please notify study recruiter)

For the purpose of this survey, "**household pets**" is loosely defined to include pets that are indoor only, outdoor only, and those that spend time both indoor and outdoor.

Questionnaire begins on the following page.

1. Do you have one or more pet cats currently in your household (this includes cats that are indoor only, outdoor only, and those that spend time both indoor and outdoor)? Yes [ ] (if ☑, please answer questions on this page)
No [ ] (if ☑, please proceed to next page)

a. How many household cats do you currently have?_________
indoor only____ # outdoor only___ # spend time both indoor and outdoor___ (on a leash outdoors? Yes [ ] No [ ])

	Yes	No	Don't Know
b. Are any of the cats declawed?	Y	N	DK
c. Have any of the cats ever bitten anyone?	Y	N	DK
d. Have any of the cats ever scratched anyone?	Y	N	DK
e. Are all the cats on a flea prevention program?	Y	N	DK
f. Are all the cats on an intestinal parasite (worm) prevention program?	Y	N	DK
g. Do any of the cats drink from the toilet bowl?	Y	N	DK
h. Are any of the cats fed in the kitchen?	Y	N	DK
i. Are any of the cats used for breeding?	Y	N	DK
j. Do all the cats visit the veterinarian at least once per year?	Y	N	DK

k. How often do any of the cats go to the bathroom outside of the litter box in the house?
Always [ ] Often [ ] (at least once per week) Sometimes [ ] (less than once per week) Never [ ]

l. How often are the litter boxes cleaned, on average?
Daily [ ] Every several days [ ] Weekly [ ] Every 2 weeks [ ] Greater than every 2 weeks [ ]

m. Please indicate which items are fed to any of the cats (check all that apply):
Commercial canned/dry food [ ]
Home cooked pet food [ ]
Home cooked human food (table scraps) [ ]
Raw meat [ ]
Raw eggs [ ]
Raw animal product treats [ ]
Commercial processed pet treats [ ]

n. How would you describe your household's emotional attachment to the cat(s)?
Very attached [ ] Somewhat attached [ ] Not very attached [ ] Not at all attached [ ]

o. If there are children under 16 years of age in your household, please answer the following questions:

	Yes	No	Don't Know
1. Do any of the children ever clean the litter box(es)?	Y	N	DK
If Yes,			
a. Do the children wear gloves when cleaning the litter box(es)?	Y	N	DK
b. Do the children wash their hands after cleaning the litter box(es)?	Y	N	DK
2. Do any of the children have access to the litter box(es)?	Y	N	DK
3. Do any of the children touch one or more of the cat(s)?	Y	N	DK

If Yes,
a. How often do any of the cats sleep in one of the children's beds?
Always [ ] Usually [] (3-6 nights/week) Sometimes [ ] (less than 3 nights/week) Never [ ]

b. Do the children wash their hands after touching the cat(s)?
Always [ ] Usually [ ] Sometimes [ ] Never [ ]

4. How would you describe the children's emotional attachment to the cat(s)? (If more than one child, how attached is the child with the closest relationship to the cat(s)?)
Very attached [ ] Somewhat attached [ ] Not very attached [ ] Not at all attached [ ]

2. Do you have one or more pet dogs currently in your household (this includes dogs that are outdoor only and those that spend time both indoor and outdoor)? Yes [ ] (if ☑, please answer questions on this page)
No [ ] (if ☑, please proceed to next page)

a. How many household dogs do you currently have?_________
outdoor only___ # spend time both indoor and outdoor___

	Yes	No	Don't Know
b. Have any of the dogs ever bitten anyone?	Y	N	DK
c. Are all the dogs on an intestinal parasite (worm) prevention program?	Y	N	DK
d. Are all the dogs on a flea prevention program?	Y	N	DK
e. Are any of the dogs used for hunting?	Y	N	DK
f. Do any of the dogs drink from the toilet bowl?	Y	N	DK
g. Are any of the dogs fed in the kitchen?	Y	N	DK
h. Are any of the dogs used for breeding?	Y	N	DK
i. Do all the dogs visit the veterinarian at least once per year?	Y	N	DK

j. Within the past 3 months, have any of your dogs been to any of the following places (check all that apply)?
On leash at a dog park [ ] Off leash at a dog park [ ] Doggie daycare or boarding [ ] Dog sporting event (e.g., flyball) [ ]

k. When outdoors, how often are the dogs either in a fenced yard or on a leash?
Always [ ] Sometimes [ ] Never [ ]

l. Please indicate which items are fed to any of the dogs (check all that apply):
Commercial canned/dry food [ ]
Home cooked pet food [ ]
Home cooked human food (table scraps) [ ]
Raw meat [ ]
Raw eggs [ ]
Raw animal product treats, such as pig's ears or rawhides [ ]
Commercial processed pet treats [ ]

m. How would you describe your household's emotional attachment to the dog(s)?
Very attached [ ] Somewhat attached [ ] Not very attached [ ] Not at all attached [ ]

n. How often do any of the dogs inappropriately go to the bathroom in the house?
Always [ ] Often [ ] (at least once per week) Sometimes [ ] (less than once per week) Never [ ]

o. If there are children under 16 years of age in your household, please answer the following questions:

	Yes	No	Don't Know
1. Do any of the children ever clean-up the dog's feces?	Y	N	DK
If Yes,			
a. Do the children wear gloves or use a scooper?	Y	N	DK
b. Do the children wash their hands after cleaning-up the dog's feces?	Y	N	DK
2. Do any of the children play in the same area as where the dog(s) go to the bathroom	Y	N	DK
a. If Yes, how often are feces removed? Immediately [ ] Daily [ ] Weekly [ ] Greater than weekly [ ]			
3. Do any of the children touch one or more of the dog(s)	Y	N	DK

If Yes,
a. How often do any of the dogs sleep in one of the children's beds?
Always [ ] Usually [ ] (3-6 nights/week) Sometimes [ ] (less than 3 nights/week) Never [ ]

b. Do the children wash their hands after touching the dog(s)?
Always [ ] Usually [ ] Sometimes [ ] Never [ ]

c. How often do any of the dogs lick one of the children's faces?
Daily [ ] Often [ ] (several times per week) Sometimes [ ] Never [ ]

4. How would you describe the children's emotional attachment to the dog(s)? (If more than one child, how attached is the child with the closest relationship to the dog(s)?)
Very attached [ ] Somewhat attached [ ] Not very attached [ ] Not at all attached [ ]

3. Do you have one or more reptiles (e.g., snake, lizard, turtle) or amphibians (e.g., frog, toad, salamander) currently in your household?

Yes [ ] (if ☑, please answer questions on this page)
No [ ] (if ☑, please proceed to next page)

a. Indicate what type(s) and number of reptiles and amphibians are currently in your household:

**Species**	**Number**
Lizard	
Turtle	
Snake	
Frog/toad	
Salamander	
Other (please list):	

	Yes	No	Don't Know
1. Are any of the animals allowed to roam freely through the house?	Y	N	DK
2. Are any of the animals allowed to roam in the kitchen?	Y	N	DK
3. Are any of the cages or animals washed in the kitchen sink?	Y	N	DK
4. Are any of the cages or animals washed in the bathtub or bathroom sink?	Y	N	DK
5. How often is the cage cleaned, on average (choose one)? Daily [ ] Every several days [ ] Weekly [ ] Every 2 weeks [ ] Greater than every 2 weeks [ ]			
6. How would you describe your household's emotional attachment to the reptile(s)/amphibian(s)? Very attached [ ] Somewhat attached [ ] Not very attached [ ] Not at all attached [ ]			

7. If there are children under 16 years of age in your household, please answer the following questions:

	Yes	No	Don't Know
a. Do any of the children ever clean the animal's cage?	Y	N	DK
If Yes,			
1. Do the children wear gloves?	Y	N	DK
2. Do the children wash their hands after cleaning the animal's cage?	Y	N	DK
b. Are any of the animals allowed to roam in the children's room?	Y	N	DK
c. Do any of the children touch any of these animal(s)?	Y	N	DK
If Yes, do the children wash their hands after touching the animals? Always [ ] Usually [ ] Sometimes [ ] Never [ ]			
d. How would you describe the children's emotional attachment to the reptile(s)/amphibian(s)? (If more than one child, how attached is the child with the closest relationship to the reptile(s)/amphibian(s)?) Very attached [ ] Somewhat attached [ ] Not very attached [ ] Not at all attached [ ]			

4. Do you have one or more pet fish currently in your household?

Yes [ ] (if ☑, please answer questions on this page)
No [ ] (if ☑, please proceed to next page)

a. How many fish do you have in your household?________

b. How often is the aquarium cleaned, on average?					
Daily [ ]	Every several days [ ]	Weekly [ ]	Every 2 weeks [ ]	Greater than every 2 weeks [ ]	
**c. Where do you dump aquarium water (check all that apply)?**					
Toilet [ ]	Bathtub or shower [ ]	Kitchen Sink [ ]	Bathroom Sink [ ]	Outside [ ]	Other [ ]
**d. How would you describe your household's emotional attachment to the fish?**					
Very attached [ ]	Somewhat attached [ ]	Not very attached [ ]	Not at all attached [ ]		

e. If there are children in your household under 16 years of age, please answer the following questions:

	Yes	No	Don't Know
1. Do any of the children ever clean the aquarium?	Y	N	DK
If Yes,			
a. Do the children wear gloves?	Y	N	DK
b. Do the children wash their hands after cleaning the aquarium?	Y	N	DK
2. Do any of the children ever touch the fish or aquarium water?	Y	N	DK
If Yes, do the children wash their hands after touching the fish or aquarium water? Always [ ] Usually [ ] Sometimes [ ] Never [ ]			
3. How would you describe the children's emotional attachment to the fish? (If more than one child, how attached is the child with the closest relationship to the fish?) Very attached [ ] Somewhat attached [ ] Not very attached [ ] Not at all attached [ ]			

5. Do you have one or more pet birds, rabbits, ferrets, hedgehogs, or rodents (such as gerbils, hamsters, guinea pigs, mice, and rats) currently in your household? Yes [ ] (if ☑, please answer questions on this page)
No [ ] (if ☑, please proceed to next page)

a. Indicate below what type(s) and number of birds, rabbits, ferrets, hedgehogs, or rodents are currently in your household:

**Species**	**Number**
Bird (indicate species):	
Rabbit	
Ferret	
Hedgehog	
Gerbil	
Hamster	
Guinea Pig	
Rat/mouse	
Other (please list):	

b. If **birds** are currently in your household, please answer the following questions:

1. How often is the cage cleaned, on average?<br>Daily [ ] Every several days [ ] Weekly [ ] Every 2 weeks [ ] Greater than every 2 weeks [ ]			
2. How would you describe your household's emotional attachment to the birds?<br>Very attached [ ] Somewhat attached [ ] Not very attached [ ] Not at all attached [ ]			
3. If there are children in your household under 16 years of age, please answer the following questions:	Yes	No	Don't Know
a. Do any of the children ever clean the bird's cage?	Y	N	DK
If Yes,			
1. Do the children wear gloves?	Y	N	DK
2. Do the children wash their hands after cleaning the bird's cage?	Y	N	DK
b. Do any of the children touch any of the birds?	Y	N	DK
If Yes,<br>1. Do the children wash their hands after touching the birds?<br>Always [ ] Usually [ ] Sometimes [ ] Never [ ]			
c. How would you describe the children's emotional attachment to the birds? (If more than one child, how attached is the child with the closest relationship to the bird(s)?)<br>Very attached [ ] Somewhat attached [ ] Not very attached [ ] Not at all attached [ ]			

c. If **rabbits, ferrets, hedgehogs, or rodents** (such as gerbils, hamsters, guinea pigs, mice, and rats) are currently in your household, please answer the following questions:

1. How often is the cage cleaned, on average?<br>Daily [ ] Every several days [ ] Weekly [ ] Every 2 weeks [ ] Greater than every 2 weeks [ ]			
2. How would you describe your household's emotional attachment to these animals?<br>Very attached [ ] Somewhat attached [ ] Not very attached [ ] Not at all attached [ ]			
3. If there are children in your household under 16 years of age, please answer the following questions:	Yes	No	Don't Know
a. Do any of the children ever clean the animal's cage?	Y	N	DK
If Yes,			
1. Do the children wear gloves?	Y	N	DK
2. Do the children wash their hands after cleaning the cage?	Y	N	DK
b. Do any of the children touch any of these animals?	Y	N	DK
If Yes, do the children wash their hands after touching these animals?<br>Always [ ] Usually [ ] Sometimes [ ] Never [ ]			
c. How would you describe the children's emotional attachment to these animals? (If more than one child, how attached is the child with the closest relationship to these animals?)<br>Very attached [ ] Somewhat attached [ ] Not very attached [ ] Not at all attached [ ]			

6. Do you currently live on a farm (e.g., have livestock, horses, or poultry on your property)?

Yes [ ] (if ☑, please answer questions on this page)
No [ ] (if ☑, please proceed to next page)

a. Please indicate the types of animals and number currently present:

**Type of animal**	**Number**
Horse(s)	
Cattle (beef)	
Cattle (dairy)	
Pig(s)	
Chicken(s)	
Duck(s)/turkey(s)	
Goat(s)	
Sheep	
Alpaca(s)/ llama(s)	
Other (list):	
Other (list):	

b. If there are children in your household under 16 years of age, please answer the following questions:

1. How often do any of the children help feed any of the animals?			
Always [ ]	Usually [ ]	Sometimes [ ]	Never [ ]
2. How often do any of the children help clean-up the animal(s) or their stalls/pens?			
Always [ ]	Usually [ ]	Sometimes [ ]	Never [ ]
3. If the children touch one or more of the farm animals or its stall/pen, do they wash their hands afterwards?			
Always [ ]	Usually [ ]	Sometimes [ ]	Never [ ]
